# Beyond Transduction: Anti-Inflammatory Effects of Cell Penetrating Peptides

**DOI:** 10.3390/molecules29174088

**Published:** 2024-08-29

**Authors:** Jack Lopuszynski, Jingyu Wang, Maliha Zahid

**Affiliations:** Department of Cardiovascular Medicine, Guggenheim Gu 9-01B, Mayo Clinic, 200 First Street SW, Rochester, MN 55905, USA

**Keywords:** cell penetrating peptides, inflammation, NF-κB, protein transduction domains

## Abstract

One of the bottlenecks to bringing new therapies to the clinic has been a lack of vectors for delivering novel therapeutics in a targeted manner. Cell penetrating peptides (CPPs) have received a lot of attention and have been the subject of numerous developments since their identification nearly three decades ago. Known for their transduction abilities, they have generally been considered inert vectors. In this review, we present a schema for their classification, highlight what is known about their mechanism of transduction, and outline the existing literature as well as our own experience, vis a vis the intrinsic anti-inflammatory properties that certain CPPs exhibit. Given the inflammatory responses associated with viral vectors, CPPs represent a viable alternative to such vectors; furthermore, the anti-inflammatory properties of CPPs, mostly through inhibition of the NF-κB pathway, are encouraging. Much more work in relevant animal models, toxicity studies in large animal models, and ultimately human trials are needed before their potential is fully realized.

## 1. Introduction

Among the most promising forefronts for novel vectors has been the development of protein transduction domains, more commonly referred to as cell penetrating peptides (CPPs). CPPs are small molecules ranging from 5 to 30 amino acids in length, which function to transduce the cell membrane either alone or attached to a variety of cargos [1]. CPPs are unique in their ability to cross the cell membrane without requiring the use of a receptor, a function first identified in 1988 in HIV’s trans-activator of transcription protein (Tat) [2,3]. Notably, the entire protein is not responsible for cell transduction; the identification of a short, 11-amino-acid, primarily cationic sequence within Tat [3], which proved to be responsible for crossing the membrane, has paved the way for the research and development of CPPs as we know them today.

Following the discovery of Tat, other non-cell specific CPPs have been discovered. This includes Antp, also called penetratin, discovered from *Drosophila melanogaster* [4]. CPPs have traditionally been used mostly for their ability to cross cell membrane barriers and deliver large or hydrophobic cargos, as first demonstrated through the conjugation of proteins such as RNase A, β-galactosidase, and horseradish peroxidase to Tat; in this case, transduction was robustly observed without any cell-type specificity [5]. Such transduction has also been observed in vivo, with protein functionality being preserved after transduction into all tissues including brain tissue [6]. Since then, CPPs, including those exhibiting cell specificity, have been used in a variety of applications including targeted drug delivery [7,8,9], the delivery of RNA interference agents such as siRNA [10,11,12,13,14], enhancing the delivery of viral vectors [15], and radioactive agents [16,17], along with many more applications [18,19,20,21,22].

While CPPs remain an exciting frontier in terms of targeted delivery and enhancing cell transduction, they have generally been considered inert vectors without any intrinsic biological properties. Compared to traditional viral vectors that require immunomodulation to gain long-term expression [23], and AAV (adeno-associated virus) vectors that can lead to immunotoxicity [24] or cytokine release syndrome [25], significant immune responses to CPPs have not been reported. Although studies on both the targeted and non-targeted delivery of potentially therapeutic or diagnostic molecules abound in the literature, the intrinsic biological properties of CPPs have received little attention. Therefore, this review will focus on the existing literature on the intrinsic biological properties of CPPs beyond transduction, which taken together could represent advantages over viral vectors, and potentially provide novel solutions to treat and prevent disease more effectively.

## 2. Mechanisms and Characteristics of CPPs

### 2.1. Basic Classification of CPPs

**Figure 1:**
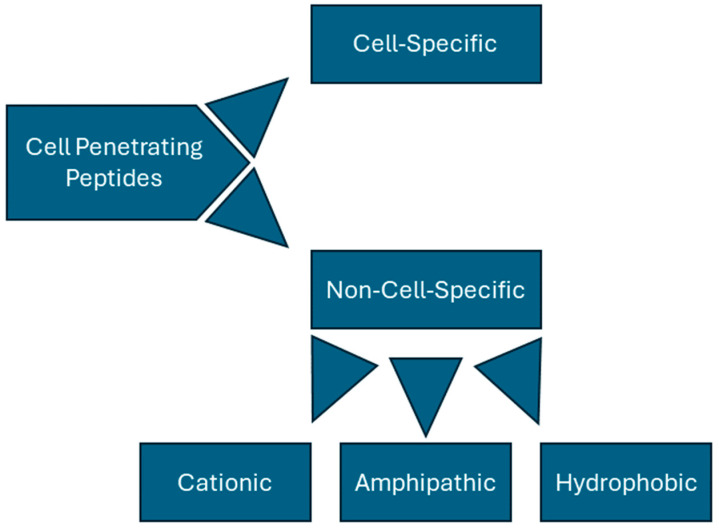
Classification for CPPs.

CPPs can be categorized in a variety of ways but are generally divided into non-cell-specific peptides and cell-specific peptides. Non-specific peptides can be further divided based on their structures and associated charges; these peptides are often classified as being cationic, hydrophobic, or amphipathic (Figure 1). Tat (YGRKKRRQRRR) and penetratin (RQIKIWFQNRRMKWKK) are key examples of cationic peptides, rich in both lysine and arginine. Learning from this observation, synthetic, non-naturally occurring peptides have been synthesized by simply using homopolymers of arginine [26] or lysine [27] residues. In fact, Wender et al. found that peptides consisting of only L-arginine or D-arginine were over 20 times better at cell transduction than Tat alone, suggesting that Tat’s cationic properties play a major role in its transduction abilities [26]. Such peptides, however, seem to have a clearly defined length; poly-arginine peptides have been found to be ineffective with less than six residues [28], and poly-lysine polymers show a decrease in transduction with greater than twelve amino acids [29]. 

Though far less common than the other types of CPPs, hydrophobic CPPs have also been successfully used for membrane transduction. These peptides are often created by the conjugation of hydrophobic signal peptide sequences, which usually function to allow the secretion of proteins from inside the cell membrane. Modifying these sequences can reverse the direction of peptide travel, and instead lead to membrane transduction [1]. Modifications to the structure of these peptides can serve to enhance their stability in solution as well as their transduction ability. One such peptide called TP10, an analog of transportan [30], has been successfully modified to enhance its three-dimensional structure and therefore its transduction abilities [31]. The stabilization of three-dimensional structures, specifically helices, has been shown to enhance the membrane transduction and delivery efficiency [32]. However, hydrophobic CPPs are less stable in solution and present many challenges when it comes to their synthesis and use, such as their tendency to aggregate due to their hydrophobic nature [33].

Amphipathic CPPs, the final type of non-specific cell penetrating peptides, are hybrid peptides created by attaching a hydrophilic sequence to a hydrophobic CPP. For instance, LAH4-L1 is an amphipathic CPP modulated with a nuclear localization signal (NLS) from Simian virus 40; this peptide was able to affect gene expression up to 10-fold compared to the baseline levels of expression [34]. Amphipathic peptides can also be created from a mixture of evenly distributed hydrophobic amino acids and other hydrophilic amino acids [35], allowing them to directly interact with the lipid bilayer for internalization [36].

CPPs can also be classified based on other properties such as the nature of their origin and functional characteristics. Gori et al. [37] have detailed a comprehensive classification system based on multiple properties; however, they admit that a fully rigid system for classifying CPPs is impossible to achieve due to the wide-ranging nature of the peptides. Using their taxonomy, CPPs are classified based on many factors including origin, structure, physical and chemical properties, specificity, and internalization mechanism. These properties tend to overlap; for instance, as discussed above, amphipathic CPPs have both synthetic and natural properties as well as distinct structural classifications (hydrophobic sequence + NLS). CPPs can be naturally occurring, totally synthetic, or a combination of both [4,37,38,39]. Other peptides were engineered to directly interact with various mechanisms of endocytosis [40]. This method of classification can be advantageous, especially in the context of drug delivery, as peptides that exhibit internalization by endocytosis must also exhibit some form of endosomal escape for effective cargo delivery. Furthermore, CPPs that interact with endocytic pathways also have the potential to effect other cellular functions through various signaling pathways. Hence, a classification of CPPs based on protein interactions can also shed light on myriad potential biological effects.

The method of discovery of CPPs can also provide a functional taxonomy for their classification. Traditionally, CPPs have been discovered using a combination of methods as described above, as well as through techniques such as phage display [1,41]. These approaches, while fruitful, are random and rely mostly on trial and error [39]. The continued discovery of CPPs combined with the rise in prevalence of bioinformatics and artificial intelligence (AI) provides another pathway for the discovery and classification of CPPs. For instance, bioinformatic approaches have been used to identify possible CPPs from the proteome of SARS-CoV-2 [42]. Machine learning algorithms such as MLCPP 2.0 [43] and DeepCPPred [44] can effectively predict both CPP sequences and uptake efficiencies. Taken altogether, the classification of CPPs is a continuous subject of study in a massive scientific field, and the readers are referred to several excellent reviews for more information on CPP classification [37,39].

### 2.2. CPP Transduction

Despite many years of research, the exact mechanism of how CPPs transduce the membrane remains elusive and likely varies between CPP subtypes, the cargoes they are carrying, and concentrations used for delivery. Furthermore, CPP internalization has been observed in both energy dependent and energy independent manners. Which pathway a given peptide uses will depend on both the properties of the cell membrane as well as the properties of the peptide and/or its cargo [45].

Initially, membrane transduction by CPPs was observed to solely occur as an energy independent process. Such transduction with transportan was observed at a range of temperatures from 0 to 37° C and was not prevented by endocytosis inhibition at high concentrations [46]. Tat and penetratin have also been observed to enter the cell by energy independent methods [4,38,39]. In an attempt to explain this phenomenon, Grasso et al. [47] described in detail how membrane mechanics are affected by different CPPs. The binding of various CPPs can rearrange lipid molecules, leading to changes in membrane stiffness and eventual peptide internalization. They also suggest that highly charged CPPs may attract more water to the lipid membrane, further destabilizing it and allowing for peptide entry [39,47]. Indeed, it has been observed that changing the physical shape of a cellular membrane can affect the transduction ability [48]. The changes in the cell membrane that result from CPP association could force the formation of inverted micelles containing the peptide, which then facilitates release into the cytoplasm [49].

Other factors also play a role in direct CPP internalization. Ionization status can affect direct membrane transduction, as described by Grasso et al. [50]. Membrane polarization can also affect peptide internalization in some cases; Wallbrecher et al. observed endocytic uptake of nona-arginine at low concentrations, but direct internalization at high concentrations due to membrane potential dependent sphingomyelinase activation [51]. Concentration and membrane composition are also vital factors in the world of CPP transduction. Walrant et al. [52] described how the membrane bound CPP fraction can be 5-fold to 100-fold higher than the internalized peptide fraction. Changing the concentration of CPPs to increase the bound peptide can cause endocytosis to occur due to increased proteoglycan clustering. Furthermore, the authors note that membrane composition plays a vital role in peptide interactions with the membrane and subsequent internalization; increased peptide–membrane binding due to differences in membrane composition can increase internalization independently of any metabotropic effects the peptide may have on the cell. 

Although energy-independent processes for CPP internalization have been widely observed, this process can also occur through endocytic (energy-dependent) pathways, particularly at lower peptide concentrations. Because endocytic pathways create endosomes, CPPs must be able to both induce endocytic uptake and escape the endosome once internalized. Although many endocytic pathways exist, macropinocytosis seems to be the prevailing mechanism for CPP transduction [45]. For instance, Ichimizu et al. [40] described how macropinocytosis facilitates the entry of a peptide called palmitoyl-cyclic-(D-Arg)12/HSA. In this study, this peptide interacted with several key macropinocytosis markers including CXCR4 and pathways such as the PKC and mTOR pathways. Endosomal escape of palmitoyl-cyclic-(D-Arg)12/HSA was also observed to be inhibited by the presence of heparin, suggesting further evidence that electrostatic interactions play a key role in endosomal escape. Interestingly, this finding suggests that the methods CPPs use to transduce cells by changing phospholipid membrane dynamics can also contribute to endosomal escape.

Other endocytic pathways besides macropinocytosis can be responsible for CPP internalization; for instance, clathrin-mediated endocytosis (CME) provides a logical method for CPP transduction. CME occurs after an endocytosis-inducing ligand binds to a receptor [45]. Therefore, it stands to reason that CPPs could possibly act as agonists to a CME-associated receptor, activating endocytosis. This phenomenon has been observed with multiple CPPs including Tat; Richard et al. observed that chlorpromazine, a known inhibitor of CME, inhibited the transduction of Tat in HeLa cells, while filipin III and nystatin, both inhibitors of caveolin-dependent endocytosis, had little to no effect on Tat’s transduction efficiency [53]. CME dependent transduction has also been observed with other CPPs. For instance, siRNA knockdown of syndecan-4, a surface proteoglycan associated with CME, has been shown to prevent the uptake of an eight oligomer of arginine [54]. Other studies have also shown chlorpromazine to be an effective inhibitor of CME-associated CPP transduction in various cell types and with various CPPs [55,56,57,58,59,60]. It does appear, however, that the primary pathway (CME, macropinocytosis, direct transduction, etc.) a peptide uses to transduce the membrane is also dependent on concentration; at lower concentrations, and especially when peptides are attached to large cargo, endocytosis is the driving factor behind peptide internalization. At higher concentrations, peptides appear to transduce the membrane independently of endocytosis [45].

### 2.3. Cell-Specific CPPs

Although non-cell-specific CPPs have played a vital role in CPP research and present exciting new ways to cross the plasma membrane, they show limited clinical usefulness for targeted cell delivery. Cell-specific CPPs provide several key advantages over non-specific CPPs, namely, requiring lower concentrations and avoiding off-target interactions and hence potential side effects. The identification of such CPPs has largely been accomplished through the use of phage display libraries [1], a technique first identified by Smith [41]. This process has been used successfully many times to identify a variety of cell-specific CPPs including CPPs to target cardiomyocytes [61], dendritic cells [62], B lymphocytes [63], pancreatic islet cells [64], and more, and does not require a priori knowledge of the internalization or binding partners before a cell-specific CPP can be identified. 

CPPs with specific biological activity present a new forefront in CPP research. While many studies have been performed to assess the toxicity of such peptides [65,66,67,68,69,70], less research exists on the intrinsic biological and/or therapeutic properties of these peptides. Cell-specific CPPs with inherent therapeutic properties present a new wave of drug development for highly targeted, direct-to-tissue therapies.

## 3. Biological Effects and Therapeutic Implications

Cell penetrating peptides have been the subject of intense research since the serendipitous discovery of the Tat protein as having intrinsic transduction ability. These studies have focused on identifying new naturally occurring or synthetic CPPs, their mechanism(s) of transduction, and their applications in both diagnostic and therapeutic arenas. To date, CPPs have largely been considered inert, small peptides with robust transduction abilities, but have been assumed to exhibit little intrinsic biological activity beyond transduction. Biological effects, other than transduction, of the CPPs presented here fall into three categories. A handful of studies have looked at the intrinsic anti-inflammatory properties of established CPPs. A second group of peptides are derived from molecular pathways associated with Toll-like receptors (TLRs), or the NF-κB pathways, which were found to also have cell transduction properties. The third, largest group are CPPs not having, or not being studied for, intrinsic anti-inflammatory properties but instead being used to deliver cargo potentially targeting various inflammatory pathways. In these studies, simply the transduction abilities of the CPP were being utilized. Our review details the work in the first two categories, and a summary of the specific biological effects of individual peptides included in this review are detailed in Table 1.

### 3.1. CPPs with Intrinsic Biological Activity

As stated above, some of the earliest CPPs were cationic and non-cell specific such as Tat, Antennapedia homeodomain-derived peptide, and synthetic homopolymers of arginine (nona-arginine). These three were compared in a head-to-head fashion in vitro in a human cervical cancer and human melanoma cell line. All three inhibited TNF-α mediated signal transduction by inducing the internalization of TNF-α receptors via clathrin mediated endocytosis [71]. The full-length Tat protein had a similar effect, and apoptosis caused by Smac protein (an inhibitor of “inhibitors of apoptosis”, or IAPs) was abrogated by Antennapedia [71]. Another in vitro study investigated the effects of various concentrations of an arginine homopolymer (octa-arginine) with incubations at various lengths of time on a U-937 macrophage cell line. There was an initial burst of super-oxide production at 30 min, but not at later time points, and no increase in the production of pro-inflammatory cytokines like TNF-α, IL-1β, or IL-6 [72]. In another study, antennapedia-derived CPP penetratin decreased the transcriptional activity of NF-κB in TNF-α stimulated L929 fibroblasts and lipopolysaccharide-activated RAW 264.7 macrophages [73]. Moreover, in an in vivo rat model of acute pancreatitis, pre-treatment with 2 mg/Kg of penetratin attenuated the severity of pancreatitis by inhibiting the IκB degradation and nuclear import of NF-κB dimers, inhibiting the expression of several downstream proinflammatory genes [73]. 

### 3.2. Anti-Inflammatory Peptides with Intrinsic Transduction Ability

In contrast to the above studies that explored the anti-inflammatory nature and pathways involved of known, well-established CPPs, the following studies aimed to design peptides with cell penetrating properties and the ability to target different inflammatory pathways, or have aimed to identify anti-inflammatory peptides that serendipitously have cell transduction properties. In this regard, the NF-κB signaling pathway, and modulation thereof, has been the subject of numerous studies due to its central role in a number of inflammatory pathologies ranging from, but not limited to, atherosclerosis, diabetes, and rheumatoid arthritis. There are highly conserved DNA-binding domains across all NF-κB members and investigators have searched for peptides that would bind to these domains, thereby inhibiting NK-κB transcriptional activities. One such peptide, anti-inflammatory peptide 6 (AIP6-RLRWR), inhibited the DNA binding and transcriptional activity of the p65 NF-κB subunit in stimulated macrophages in vitro, and inhibited zymosan-induced inflammation in vivo in mice [74]. In other studies, another bifunctional peptide, cSN50.1 (AAVALLPAVLLALLAPCVQRKRQKLMPC), with the molecular wight of 2986 Da, was designed to inhibit the nuclear transport of stress-mediated transcription factors [75]. This peptide mimics the NF-κB/p50 nuclear localizing sequence, with further studies showing that it selectively targets importin α5 [84] and mitigates atherosclerosis, fatty liver, blood glucose, and lipid levels in a mouse model of familial hypercholesterolemia [85]. This peptide has a hydrophobic region (AAVALLPAVLLALLAP) and an arginine/lysine rich nuclear localizing region (CVQRKRQKLMPC); its ability to cross nuclear membranes also translates into a similar ability to cross plasma membranes. 

Anti-inflammatory cell penetrating peptides also have applications for the nervous system. Gliomas are intracranial tumors with high mortality and limited options for treatment due to the blood–brain barrier preventing achievement of adequate drug concentrations in tumor tissue. Additionally, NF-κB activation has been correlated with tumor drug resistance. To counter both of these issues, a peptide was specifically designed to have both cell penetrating and NF-κB inhibition capabilities and was conjugated to pegylated liposomes loaded with doxorubicin for delivery to glioma tumors in vivo. This approach decreased the tumor size and significantly prolonged survival in nude mice bearing intracranial gliomas [76]. Finally, a heparin-binding sequence derived from the heparin-binding epidermal growth factor transduced RAW 264.7 macrophages within ten minutes. Pretreatment with this peptide reduced the LPS-induced production of nitric oxide, inducible nitric oxide synthase, and cytokines TNF-α and IL-6, in a dose-dependent manner by blocking the phosphorylation and degradation of IκBα, leading to inhibition of nuclear translocation of the p65 subunit of NF-κB. It also decreased infiltration by polymorphonuclear cells in an in vivo lung inflammation model [77].

Eosinophil mediated inflammation plays a key role in several common allergic lung pathologies including asthma. Eosinophil granules have several proteins including eosinophil cationic protein (ECP), which is thought to play a central role in airway hyper-responsiveness. A 10-amino acid peptide (NYRWRCKNQN) derived from ECP and named CPPecp has a heparan-sulfate binding core. Several glycosaminoglycans present on cell surfaces bind to ligands to mediate myriad immune responses; therefore, it was hypothesized that CPPecp binding to these cell surface receptors will block this interaction and modulate immune responses in various models of lung inflammation. Indeed, this turned out to be true in BEAS-2B, a lung epithelial cell line, where CPPecp decreased ECP mRNA expression, eotaxin secretion, and p-STAT6 activation [78]. In vivo studies also reduced mite-induced airway inflammation by decreasing the neutrophil and eosinophil count in broncho-alveolar lavage fluid, and decreased IL-5, IL-13, IL-17, and eotaxin expression in lung tissue [78]. Further studies identified CPPecp’s anti-inflammatory activity through inhibition of the NLRP3 inflammasome [79]. 

Acute lung injury (ALI) is a life-threatening, rapidly progressing inflammatory condition driven by TLR4 activation. In an attempt to mitigate the effects of ALI, a decoy CPP designed to inhibit the binding of TLR4 to MyD88 was created and termed TM6 (RQIKIWFQNRRMKWKKENFLRDTWCNFQFY). In a mouse model of lipopolysaccharide (LPS)-induced lung injury, treatment with TM6 alleviated negative histological changes, inhibited myeloperoxidase activity, and lowered the TNF-α, IL-1β, and IL6 levels in lung tissue [80]. Inflammation via TLR4 activation also plays a key pathophysiological role in insulin resistance in type II diabetes mellitus. The death domain of MyD88 has been found to interact with TLR4. Several studies have identified a 10-residue amino acid (M10-RRRLSLFLNV) derived from the death domain of MyD88 to have cell transduction abilities (likely due to its arginine rich residues), which also functioned to inhibit the LPS-induced nuclear translocation of NF-κB, decreased TNF-α and IL6 levels, lowered blood glucose levels, and improved glucose intolerance in db/db mice [81]. 

CIBG-552 is an anti-tumor peptide with cell penetrating activity developed from the screening of an Ala-library derived from the *Limulus* anti-LPS factor (LALF) 32–51 region [86]. It has been shown to exert anti-angiogenic and anti-inflammatory effects through inhibition of the NF-κB and HIF-1 pathways via its interaction with COMMD1 [82]. Interestingly, this peptide has been tested in a Phase I clinical trial of 25 patients with advanced, refractory solid tumors [83]. Patients received varying doses of subcutaneous injections of CIBG-522 3x/week for two weeks, in which the maximum tolerated dose was found to be 4.7 mg. Stable disease was reported in five patients, and seven out of ten assessed patients showed a significant change in the ratio of CD4/CD8 cells in response to therapy [83].

### 3.3. CPPs to Deliver Anti-Inflammatory Cargo

As far as the transduction abilities of CPPs are concerned, numerous studies have utilized these properties to deliver small molecules, or other decoy peptides inhibiting various inflammatory pathways like NF-κB [87,88,89,90,91,92,93,94], the TLR4 pathway [74,95,96,97], and STAT-6 [98,99], to name a few. This list is by no means exhaustive, and we apologize to the authors whose valuable work was inevitably left out due to space limitations.

### 3.4. Anti-Inflammatory Potential of Cardiac Targeting Peptide, a CPP

**Figure 2:**
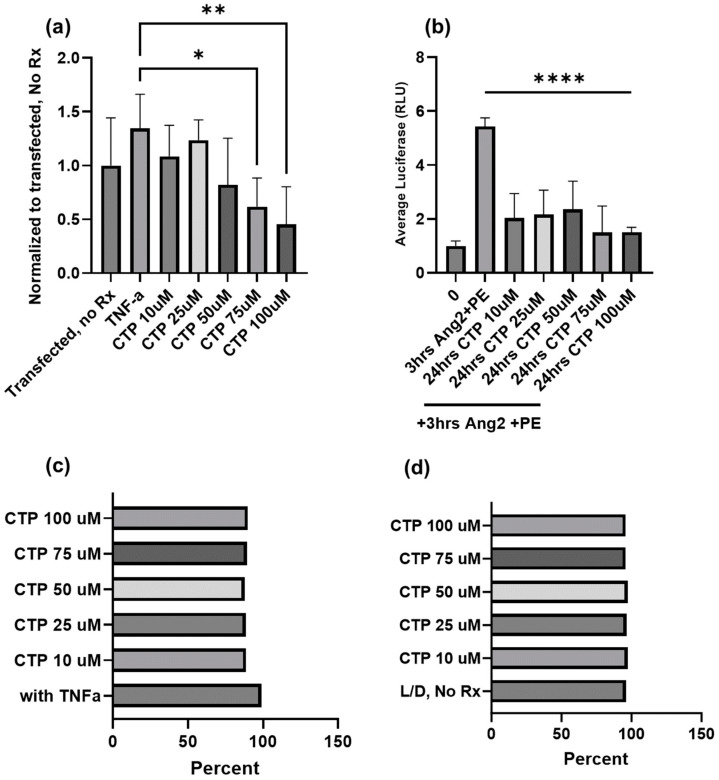
Inhibition of TNF-α mediated NF-κB activation by CTP in a human cardiomyocyte cell line at the baseline (**a**) and after angiotensin/phenylephrine challenge (**b**). No change in cell viability due to either treatments with CTP (**c**) or treatments with CTP after an angiotensin/phenylephrine challenge (**d**) as assessed by FACS using live/dead stain. *: *p* < 0.05, **: *p* < 0.01, ****: *p* < 0.0001.

We have had a long-standing interest in cell-type specific CPPs [100], and recently observed our cardiac targeting peptide (CTP: APWHLSSQYSRT) to have intrinsic biological properties beyond transduction. Our work, using a combinatorial in vitro and in vivo phage display methodology [61], identified CTP, which is a cardiomyocyte-specific CPP that transduces heart tissue in as little as 15 min after peripheral intravenous injection in mice [101]. This work has been confirmed by at least three different independent investigators from around the world [102]. Our recent work involved conjugating the N-terminus of CTP to amiodarone, a well-established anti-arrhythmic in routine clinical use via a disulfide bond [103]. The conjugate was injected intraperitoneally into guinea pigs, and after 5 days of daily injections, the animals were euthanized and their hearts placed in a Langendorff perfusion system to measure heart rates, calcium currents, and action potential durations. To our surprise, CTP alone without any cargo displayed several salutary effects on calcium handling and increased heart rates while decreasing the action potential durations [8]. RNA sequencing from these heart extracts revealed that CTP increased alpha-adrenergic receptor expression and decreased beta-adrenergic receptor expression, explaining the changes in heart rate. It also upregulated calcium handling genes (SERCA2a) and surprisingly downregulated several NF-κB pathway genes as well as TNF-α [8]. To further study the physiological effects of CTP, we used a human cardiomyocyte cell line transfected with a reporter plasmid expressing the luciferase gene under an NF-κB promoter. Post-transfection, cells were treated with varying concentrations of CTP and then challenged with TNF-α (20 ng/mL) prior to running a luciferase assay. Cell viability in response to the above manipulations was assessed by FACS using live/dead stain. CTP inhibited TNF-α mediated NF-κB activation in a dose-dependent manner, with significant inhibition seen with CTP at concentrations of 75 µM and above (*p* < 0.05; Figure 2). Furthermore, challenging cardiomyocytes with angiotensin/phenylephrine resulted in a significant increase in NF-κB activation, an increase prevented by pre-treatment with CTP. Studies looking at the molecular pathways leading to this NF-κB inhibition as well as the expression of downstream cytokines are ongoing. 

## 4. Conclusions

The potential of CPPs as novel vectors has been an area of intense study for the last three decades. They have generally been considered inert vectors for the delivery of other diagnostic or therapeutic drugs ranging from small molecules, other peptides of therapeutic potential, to oligonucleotides for gene therapy, as well as most recently RNA-interference therapeutics. Little attention has been paid to the possibility of these CPPs having inherent biological properties beyond transduction. Many of the earliest traditional non-specific CPPs like Tat and penetratin, and more recently our cardiomyocyte-targeting peptide CTP, have shown immense potential as anti-inflammatory agents, which can serve as a stand-alone therapeutic or add to the therapeutic potential of these CPPs. This is of particular significance, as the more traditional viral vectors are limited by pre-existing immunity [23] or the development of rapid immunity to these vectors on first exposure [24]. Beyond developing immunity, these viral vectors also suffer from inciting an acute inflammatory response that can be life-threatening [25] including myocarditis [104] and/or a sepsis-like syndrome [105]. In this review, we make a case for devoting more research to the less obvious, but perhaps equally important biological effects such as transduction that these CPPs exhibit. At a minimum, evolving data indicate that these peptides do not incite an immune response, a highly desirable characteristic of a vector. However, far more longitudinal in vivo studies in large animal models are necessary to confirm these findings.

## Figures and Tables

**Table 1:** A summary of the specific biological activities of various cell penetrating peptides.

CPP Name	Sequence	Function	Basic Classification
Tat [71]	Fluo-YGRKKRRQRRR-CONH_2_	Inhibit cell apoptosis	Cationic
Smac-Antp [71]	AVPIAQK-RQIKIWFQNRRMKWKK-ϵK(Fluo)-CONH_2_	Inhibit cell apoptosis	Cationic
Octa-arginine (R8) [72]	RRRRRRRR	Exposing U-937 macrophages to R8 generated superoxide anion and affected gene expression	Cationic
Penetratin [73]	RQIKIWFQNRRMKWKK	Decreased transcriptional activity of nuclear factor-κB (NF-κB) and nuclear import of NF-κB dimers, inhibiting the expression of several downstream pro-inflammatory genes	Cationic
AIP6 [74]	RLRWR	A promising lead structure for the development of specific NF-κB inhibitors as potential anti-inflammatory agents	Cationic
cSN50.1 [75]	AAVALLPAVLLALLAPCVQRKRQKLMPC	Calming the LPS-triggered “genomic storm” attenuates the systemic inflammatory response associated with lethal shock as well as localized lung inflammation	Hydrophobic
CB5005 [76,77]	KLKLALALALALAVQRKRQKLMPC	A cell-penetrating peptide and an NF-κB inhibitor	Hydrophobic
CPPecp [78,79]	NYRWRCKNQN	Decreased ECP mRNA expression, eotaxin secretion, p-STAT6 activation, and inhibition of the NLRP3 inflammasome	Cationic
TM6 [80]	RQIKI-WFQNRRMKWKKENFLRDTWCNFQFY	Alleviated negative histological changes inhibited myeloperoxidase activity, and lowered TNF-α, IL-1β, and IL6 levels in lung tissue	Hydrophobic
MyD88 [81]	M10-RRRLSLFLNV	Attenuates inflammation and insulin resistance and improves glucose metabolism	Amphipathic
CIBG-552 [82,83]	Ac-HARIKpTFRRlKWKYKGKFW	Anti-inflammatory and anti-angiogenic effects	Amphipathic

## Data Availability

Not applicable.
